# Outcomes >30 Years After Initial Nonoperative Treatment of Anterior Cruciate Ligament Injuries

**DOI:** 10.1177/03635465231214423

**Published:** 2024-01-09

**Authors:** Clara Hellberg, Ioannis Kostogiannis, Alexandros Stylianides, Paul Neuman

**Affiliations:** † Clinical Epidemiology Unit, Department of Orthopaedics, Clinical Sciences Lund, Lund University, Lund, Sweden; ‡ Department of Orthopaedics, Clinical Sciences Lund, Lund University, Lund, Sweden; § Musculoskeletal Radiology Section, Skåne University Hospital, Lund University, Lund, Sweden; ‖ Department of Orthopaedics, Clinical Sciences Malmö, Lund University, Malmö, Sweden; Investigation performed at Department of Orthopaedics, Clinical Sciences Malmo«, Lund University, Malmo«, Sweden

**Keywords:** anterior cruciate ligament (ACL), long-term follow-up, nonoperative treatment, osteoarthritis (OA)

## Abstract

**Background:**

It is unclear how anterior cruciate ligament (ACL) reconstruction (ACLR) affects the development of osteoarthritis (OA). This uncertainty is partly caused by the lack of long-term studies on ACL injuries treated primarily without reconstruction and the underreporting of symptomatic OA.

**Purpose:**

To determine (1) the knee function, symptoms, and activity level, as well as the presence of radiographic and symptomatic OA; (2) how these clinical outcomes have changed over time; and (3) the frequency of subsequent knee surgeries after the index ACL injury in a cohort of patients with ACL injuries treated primarily without reconstruction.

**Study Design:**

Case series; Level of evidence, 4.

**Methods:**

A total of 100 patients underwent initial nonoperative treatment >30 years ago (mean, 33.2 ± 1.4 years). Of these, 81 patients (mean age, 59 ± 8 years) completed the Knee injury and Osteoarthritis Outcome Score (KOOS), Lysholm Knee Scoring Scale, and Tegner Activity Scale. Seventy-three patients underwent radiography to evaluate tibiofemoral and patellofemoral OA in the ACL-injured knee. Patients only underwent late ACLR if they experienced insufficient knee stability.

**Results:**

At 33 years after the ACL injury, the KOOS Activities of Daily Living subscore was better than population-based reference values, but scores were similar for the remaining KOOS subscales. Furthermore, 65% of patients had a good or excellent Lysholm score (≥84 points). The Tegner score decreased 4 points from before the injury to 33-year follow-up (*P* < .001). Most patients (75%) had evidence of radiographic tibiofemoral and/or patellofemoral OA, but only 38% were classified as having symptomatic OA (defined as radiographic OA in combination with a symptomatic knee according to cutoffs on the KOOS). Approximately 50% underwent meniscal surgery, and 29% subsequently underwent ACLR for recurrent instability. There were 2 patients who underwent total knee replacement.

**Conclusion:**

Despite a high prevalence of radiographic OA, patients achieved acceptable subjective knee function and had a relatively low prevalence of symptomatic OA at >30 years after an ACL injury when an initial nonoperative treatment strategy was employed.

Between 100,000 and 200,000 Americans sustain an anterior cruciate ligament (ACL) injury every year,^25^ with young patients being overrepresented.^24^ An ACL injury is a well-known risk factor for developing knee osteoarthritis (OA), but it is uncertain whether ACL reconstruction (ACLR) can lower the risk of developing OA.^7,11,14,19,24^ Between 25% and 65% of all ACL ruptures have a combined meniscal injury, which further increases the risk of developing OA.^24^ A lower prevalence of secondary meniscal injuries has been reported in patients after ACLR compared with those treated nonoperatively,^11,19^ and secondary analysis of magnetic resonance imaging data from a randomized controlled trial also suggested a lower risk of subsequent meniscal damage after primary reconstruction.^21^

Although OA may develop over several decades after an ACL injury and many patients do not undergo reconstruction, few studies have investigated the development of OA in nonoperatively treated patients at >30 years after the injury. However, one study reported a prevalence of radiographic OA of up to 75% at 32 to 37 years after the injury in patients with ACL injuries treated without ACL surgery.^10^ Furthermore, most previous studies have focused only on radiographic OA changes after an ACL injury. However, as radiographic findings and symptoms are often poorly related,^2^ it is important to study clinically relevant, that is, symptomatic, OA^10,13^ because this reflects the disease burden and the need for a major surgical intervention.

In view of this, long-term follow-up studies on patients with ACL injuries who did not initially undergo reconstruction are required. Thus, the main aim of this study was to determine the knee function, symptoms, and activity level as well as the prevalence of radiographic and symptomatic OA many years after an acute ACL injury among patients primarily treated without ACLR. In addition, changes over time in these parameters were investigated. Finally, the frequency of subsequent surgery on the ACL-injured knee during the follow-up period was assessed. Our hypothesis was that patients would achieve satisfactory knee function and activity levels, despite a high prevalence of radiographic OA, at >30 years after an acute ACL injury with initial nonoperative treatment.

## Methods

### Patients

Between 1985 and 1989, a total of 200 patients with acute knee sprains in combination with hemarthrosis and/or knee instability on manual testing were referred from an emergency clinic to the Department of Orthopaedics at Skåne University Hospital, Sweden, for further evaluations. All patients included in the study were examined by the same knee specialized orthopaedic surgeon; patients who visited the clinic when he was off duty were unaccounted for (n = 85). The patients underwent diagnostic arthroscopic surgery within 10 days of the injury and were consecutively included in the study if they had a complete tear of the ACL and were aged between 15 and 45 years. Exclusion criteria were a Tegner score of >9 (n = 5); a distinct wish to undergo primary ACLR (n = 3); and previous injuries to the lower extremities, skeletal lesions on radiography, or a diagnosis of a psychosocial disorder (n = 7). The characteristics of the 100 patients eligible for inclusion are described in Table 1. During arthroscopic surgery, patients were diagnosed with either an isolated tear of the ACL or combined knee injuries of the collateral ligaments, menisci, and cartilage. None of the included patients underwent ACLR during the index arthroscopic examination. However, all combined injuries to the knee were treated according to current recommendations. Therefore, 25 patients underwent partial meniscectomy for a major meniscal injury, and 35 patients had a minor meniscal lesion that was left in situ. No collateral ligament injuries underwent reconstruction, but grade 2 to 3 injuries were treated with a brace for approximately 6 weeks. No isolated ACL injuries were treated with a brace. Furthermore, no meniscal repair was performed during index arthroscopic surgery.

**Table 1:** Patient Characteristics

	Time of Injury (n = 100)	15-y Follow-up (n = 93)	33-y Follow-up (n = 81)
Age, mean ± SD (range), y	26 ± 8 (15-43)	42 ± 8 (30-60)	59 ± 8 (48-76)
Female sex, n (%)	42 (42)	38 (41)	37 (46)
Body mass index, mean ± SD	23 ± 1.0 (n = 100)	26 ± 4.3 (n = 87)	26 ± 4.1 (n = 76)
Right leg injured, n (%)	51 (51)	47 (51)	38 (47)

All patients were followed up repeatedly and were initially treated with physical therapy. The majority attended supervised training sessions twice a week for 5 to 8 months, and the remainder underwent self-monitored training. The exercises were focused on obtaining functional knee stability, and patients were advised to avoid contact sports such as soccer and handball, as pivoting sports activities were considered a substantial risk for further knee injuries. Further information regarding the treatment algorithm, enrollment process, patient characteristics, additional knee injuries besides the ACL tear, and surgical procedures performed from the time of the ACL injury up to 15 years of follow-up is described in previous studies on the same cohort by Kostogiannis et al^9^ and Neuman et al.^16^ These studies also include data on knee function, symptoms, activity levels, and OA development up to 15 years after the injury.^9,16^

Late ACLR was recommended if patients experienced frequent knee instability with episodes of “giving way,” still had a subjectively insufficient and unacceptable activity level after the completion of physical therapy, sustained a meniscal tear that needed suture repair, or had >1 significant reinjury leading to arthroscopic meniscal surgery and would not accept a further prophylactic decrease in their activity level.

### Follow-up

**Figure 1.**
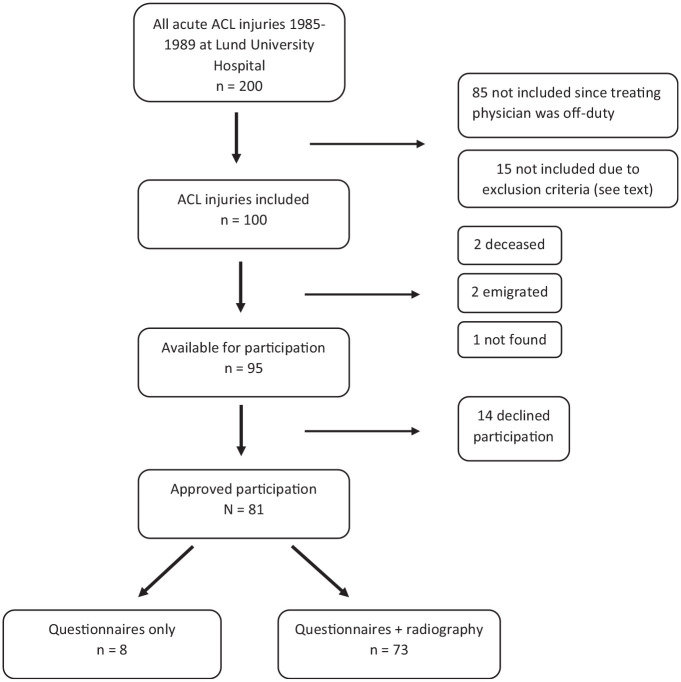
Flowchart of patient recruitment at 33-year follow-up. ACL, anterior cruciate ligament.

Long-term follow-up was performed at 31 to 35 years after the ACL injury. The mean time to final follow-up was 33 years (referred to as “33-year follow-up” hereafter). There were 95 patients available (2 patients were deceased, 2 patients had emigrated, and 1 patient's address was not found), of whom 81 were willing to complete the Knee injury and Osteoarthritis Outcome Score (KOOS), Lysholm Knee Scoring Scale, and Tegner Activity Scale online between February and July 2020. Overall, 73 patients agreed to undergo radiography of the ACL-injured knee (Figure 1), which was performed up to 1 year after the completion of the questionnaires (delay due to COVID-19).

### Ethics

The study was approved by the Regional Research Ethics Board (2019-05032; year 2019) and the Radiation Protection Committee of Region Skåne (SSM2019-8950; year 2019). All patients were invited to participate in the study via an informative letter, and those who chose to participate provided written informed consent. All data were de-identified to ensure integrity.

### Patient-Reported Outcome Measures

The patients received an invitation via email, directing them to the digital questionnaire platform REDCap (Research Electronic Data Capture; Version 6.10.11; hosted at Lund University), and were asked to complete the KOOS, Lysholm Knee Scoring Scale, and Tegner Activity Scale. The same patient-reported outcome measures (PROMs) are also available from 15-year follow-up, and for the Tegner score, additional values preceding the ACL injury are available.^9,16^

The KOOS is used to assess a patient's knee function after an injury that may result in OA. The instrument consists of 5 subscales: Pain, Symptoms, Activities of Daily Living (ADL), Sport and Recreation (Sport/Rec), and Quality of Life (QOL). The score ranges from 0 to 100 (0 = extreme problems; 100 = no problems).^20^ The tool has high validity and test-retest reliability.^3^ Our KOOS scores were compared with population-based reference values^18^ and with scores from another cohort of patients with ACL injuries treated primarily without reconstruction.^10^ Cutoffs for a symptomatic knee were applied according to a previous definition by Englund et al^4^: “This operational definition is aimed at identifying individuals symptomatic enough to possibly seek medical care. . . . After conversion to a 0-100 scale (0 = worst, 100 = best), the cutoffs were as follows: pain ≤86.1, symptoms ≤85.7, ADL ≤86.8, Sport/Rec ≤85.0, and QOL ≤87.5.” If a patient scored equal to or under the cutoff on the KOOS QOL subscale, as well as on at least 2 of the other subscales, the patient was classified as symptomatic.^4^ To determine whether there was a clinically significant difference in the scores between 15- and 33-year follow-up, we used a minimal clinically important difference of 8 points.^20^

The Lysholm Knee Scoring Scale is used to assess a patient's subjective knee function after injuries such as ACL injuries. The score ranges from 0 to 100, with <65 points considered a poor outcome; 65-83, a fair outcome; 84-94, a good outcome; and ≥95, an excellent outcome.^22^

The Tegner Activity Scale is used to measure the activity level in patients with different types of knee injuries. The score ranges from 0 to 10, with 0 to 3 points representing activities of daily living; 4 to 6 points, recreational sports and active individual sports; and 7 to 10 points, activities, with high demands on the knee.^22^

### Data Collection From Registries and Medical Records

All patients were asked to provide information on previous surgical procedures on the knee. If the patient underwent additional surgery after the index arthroscopic examination, further information regarding the type of injury and surgical methods was obtained from the Swedish National Knee Ligament Registry, the Swedish Knee Arthroplasty Register, and individual medical records.

### Radiographic and Symptomatic OA

Radiographs were obtained in standing, weightbearing, and anteroposterior views with both knees in 20° of flexion to examine the tibiofemoral (TF) joint and in the skyline view with both knees in 50° of flexion to examine the patellofemoral (PF) joint. The radiographs were evaluated by a radiologist (A.S.) and a senior orthopaedic knee surgeon (P.N.) with no access to clinical details, except for visible traces of ACLR in some cases. All radiographs were re-evaluated by both examiners jointly, and a final classification, agreed upon in consensus, was used. The images were evaluated for joint space narrowing (JSN) and osteophytes on a 4-point scale (0-3; 0 = without signs of JSN or bony changes) according to the Osteoarthritis Research Society International atlas.^1^ Radiographic TF or PF OA was considered as present if ≥1 of the following criteria was positive in either the lateral or medial TF or PF compartment: grade ≥2 of JSN, grade ≥2 of the sum of marginal osteophytes in the same compartment, or grade 1 of JSN together with 1 osteophyte in the same compartment. This represents approximately grade 2 for knee OA according to the Kellgren and Lawrence scale.^8^ To define symptomatic OA, we stratified patients into 2 groups, symptomatic or nonsymptomatic, based on the KOOS, as previously described, in combination with the presence of radiographic TF OA, PF OA, or TF and/or PF OA. In the case of radiographic OA and a symptomatic knee according to the KOOS, the patient was classified as having symptomatic OA.

### Statistical Analysis

Statistical analyses were performed using Stata/SE (Version 17.1; StataCorp). The Student *t* test was employed to calculate differences between 2 populations (comparing KOOS scores for our cohort with reference values as well as with scores from another cohort with ACL injuries), and the paired Student *t* test was used to assess differences between 15- and 33-year follow-up (KOOS and Lysholm). We used a linear mixed model to assess differences over time between before the injury, 15-year follow-up, and 33-year follow-up (Tegner). We conducted linear regression analysis, adjusted for sex, body mass index, and age at 33-year follow-up, to calculate differences on all PROM scores between groups with or without radiographic OA, with residual plots used for model diagnostics. All tests were 2-tailed. A *P* value ≤.05 was considered statistically significant, and 95% CIs are reported.

## Results

At 33-year follow-up, patients had a mean age of 59 ± 8 years, and 46% were female (Table 1). The mean time from the ACL injury to follow-up was 33.2 ± 1.4 years (range, 31-35 years).

### Loss to Follow-up

Slightly more male than female patients were lost to 33-year follow-up, while differences in age and baseline physical activity were negligible (data not shown). Compared with 15-year follow-up, slightly more patients who had undergone meniscal surgery (3 percentage points) or ACLR (1 percentage point) were lost to 33-year follow-up (Appendix Figure A1, available in the online version of this article).

### Knee Function, Symptoms, and Activity Level at 33-Year Follow-up

#### KOOS Score

**Figure 2.**
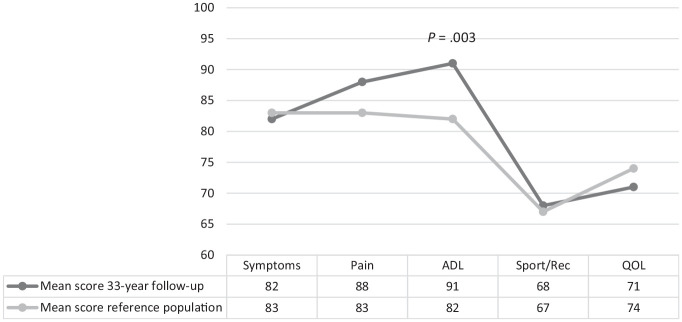
Mean Knee injury and Osteoarthritis Outcome Score subscores at 33-year follow-up and age-representative population-based reference values.^18^ At 33-year follow-up: Symptoms (n = 78), Pain (n = 78), Activities of Daily Living (ADL; n = 78), Sport and Recreation (Sport/Rec; n = 77), and Quality of Life (QOL; n = 77) (excluding patients with total knee replacement and osteotomy). Reference population: Symptoms (n = 173), Pain (n = 173), ADL (n = 173), Sport/Rec (n = 171), and QOL (n = 173).

At 33-year follow-up, 81 patients had completed the KOOS Symptoms, Pain, and ADL subscales, and 80 patients had completed the Sport/Rec and QOL subscales. Overall, patients scored high on all KOOS subscales at 33 years after their ACL injury. Compared with age-representative population-based reference values (range, 55-74 years), our patients (range, 48-76 years) scored clinically and statistically significantly better on the KOOS ADL (mean difference, 9 points [95% CI, 2.9-14.4]; *P* = .003) and similarly on the remaining subscales (Figure 2) (Appendix Table A1, available online).^18^

When stratifying the patients at 33-year follow-up as being symptomatic or nonsymptomatic according to the KOOS, 44% of patients (36/81) were symptomatic. Patients who underwent total knee replacement (TKR) and osteotomy were classified as symptomatic on the KOOS, as they had undergone surgery because of OA.

Linear regression analyses showed that patients with radiographic OA in the TF and/or PF compartments had clinically lower KOOS scores on all subscales (the difference in Symptoms subscores was not statistically significant), besides ADL subscores that were comparable with patients without radiographic OA. The largest differences were seen for the KOOS Sport/Rec and QOL (Figure 5A). Furthermore, KOOS scores were lower for patients with radiographic PF OA compared with patients with radiographic TF OA (Appendix Table A2, available online).

#### Lysholm Knee Score

**Figure 3.**
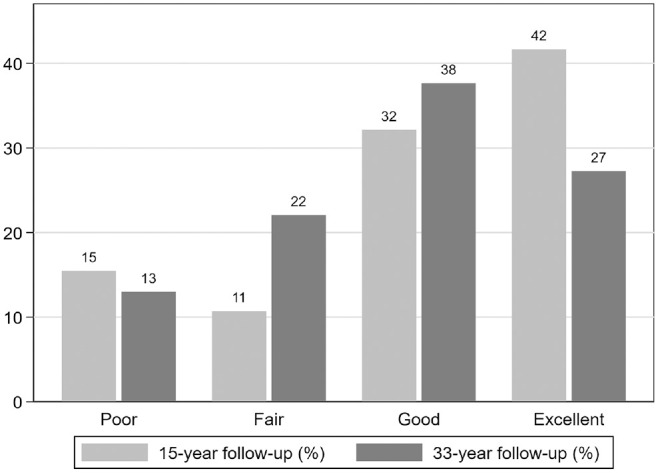
Distribution of Lysholm scores at 15- and 33-year follow-up. At 15-year follow-up: n = 84. At 33-year follow-up: n = 77 (excluding patients with total knee replacement and osteotomy).

At 33-year follow-up, 80 patients had completed the Lysholm Knee Scoring Scale. Most patients (65% [n = 50/77]) had a good or excellent Lysholm score, and only 13% (n = 10/77) had a poor Lysholm score (Figure 3). The mean score was 84 ± 17, and the median score was 89 (range, 18-100).

Linear regression analyses showed that patients with radiographic OA in the TF and/or PF compartments had statistically significantly lower Lysholm scores compared with patients without radiographic OA (Figure 5A). Patients with radiographic PF OA had lower Lysholm scores compared with patients with radiographic TF OA (Appendix Table A2, available online).

#### Tegner Activity Score

**Figure 4.**
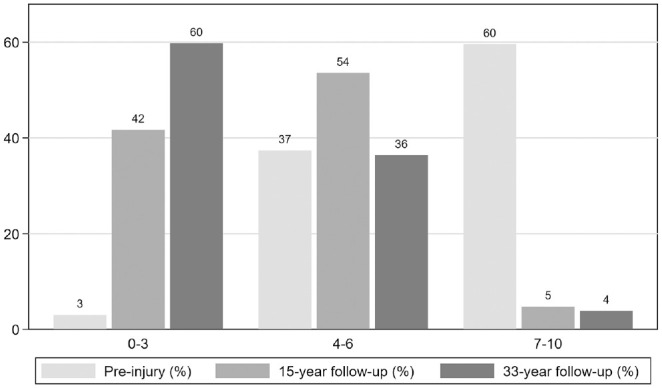
Distribution of Tegner scores before the injury and at 15- and 33-year follow-up. Before injury: n = 99. At 15-year follow-up: n = 84. At 33-year follow-up: n = 77 (excluding patients with total knee replacement and osteotomy).

At 33-year follow-up, 80 patients had completed the Tegner Activity Scale. Most patients (60% [n = 46/77]) had a Tegner score of 0 to 3 points (Figure 4). The mean score was 3 ± 2, and the median score was 3 (range, 1-7).

**Figure 5.**
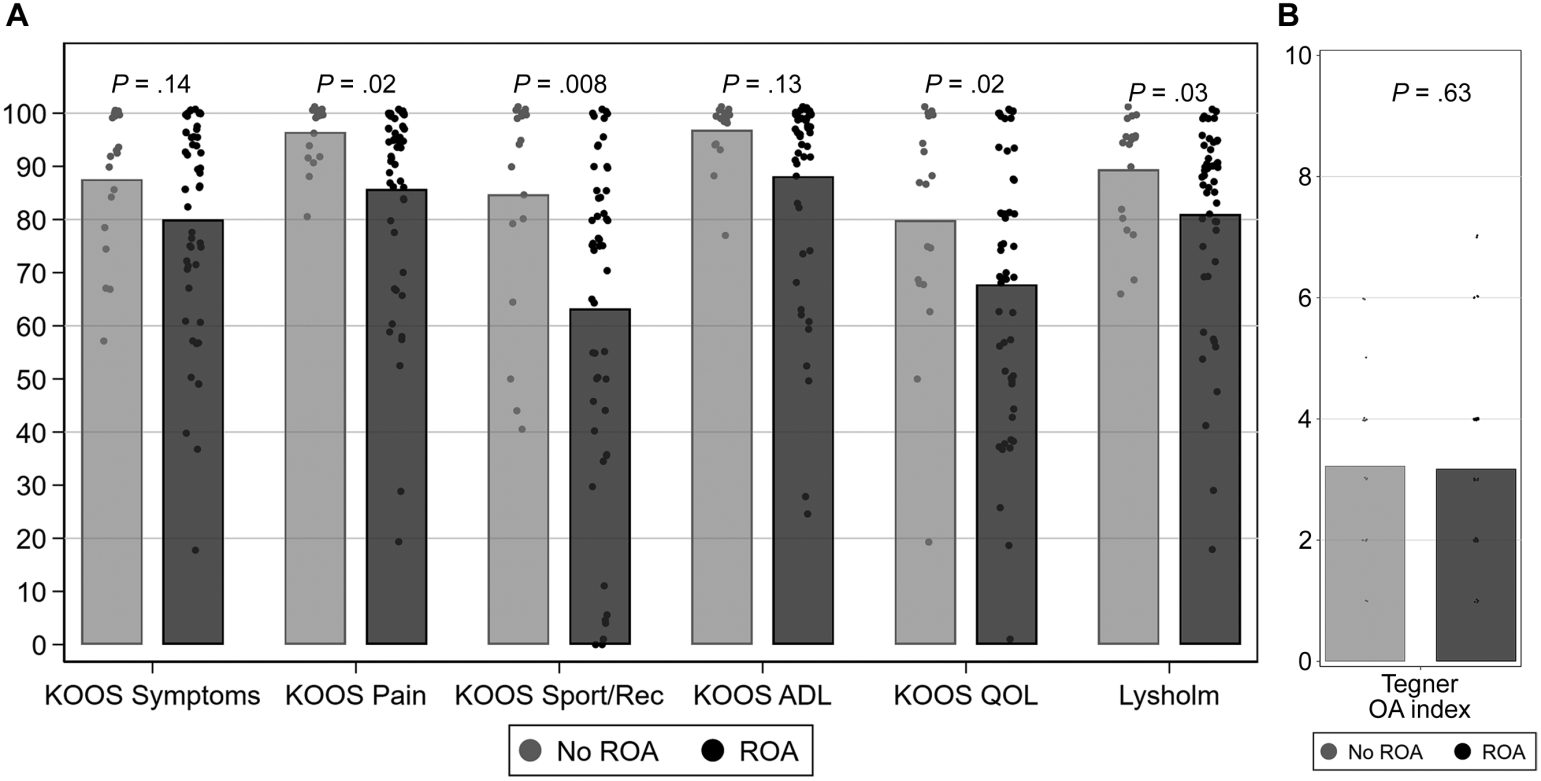
(A) Mean Knee injury and Osteoarthritis Outcome Score (KOOS) subscores and Lysholm scores, including *P* values, according to presence of radiographic osteoarthritis (ROA). n = 70 (excluding patients with total knee replacement, with osteotomy, and without radiographs). (B) Mean Tegner scores, including *P* values, according to presence of ROA. n = 70 (excluding patients with total knee replacement, with osteotomy, and without radiographs). ADL, Activities of Daily Living; KOOS, Knee injury and Osteoarthritis Outcome Score; OA, osteoarthritis; QOL, Quality of Life; Sport/Rec, Sport and Recreation.

Linear regression analyses did not show a difference in Tegner scores between patients with and without radiographic OA (Figure 5B) (Appendix Table A2, available online).

### Radiographic and Symptomatic OA at 33-Year Follow-up

A total of 73 patients had plain radiographs at 33-year follow-up. There were 2 patients who were treated with TKR. One of them was classified as positive for radiographic OA in both the TF and the PF compartments; the other did not have a patellar prosthesis, was considered positive for radiographic OA in the TF compartment, and was examined for radiographic OA in the PF compartment. One patient was treated with osteotomy and was examined for radiographic OA in both compartments. Radiographs revealed that 48 patients (66%) had radiographic TF OA, 38 patients (52%) had radiographic PF OA, and 55 patients (75%) had radiographic TF and/or PF OA (n = 73). The prevalence of radiographic OA increased with age (Appendix Table A3, available online). Patients with symptomatic OA in the TF, PF, or TF and/or PF compartments totaled 26 (36% [95% CI, 26%-47%]), 23 (32% [95% CI, 22%-43%]), and 28 (38% [95% CI, 28%-50%]), respectively (n = 73). Appendix Table A4 (available online) includes data on additional proportions. In the group with radiographic TF and/or PF OA, patients were somewhat older, and a larger proportion underwent ACLR (24% vs 11%, respectively) or meniscal surgery (49% vs 28%, respectively) during follow-up after the index ACL injury compared with patients without radiographic OA (Appendix Table A5, available online).

### Changes in Knee Function, Symptoms, and Activity Level Over Time

#### KOOS Score

Most KOOS subscores showed a nonstatistically and nonclinically significant slight decrease between 15- and 33-year follow-up. The KOOS Sport/Rec showed the largest decrease and was the only subscale that had both a statistically and clinically significant mean difference (≥8 points) according to previously described cutoffs (Table 2).

**Table 2:** Knee injury and Osteoarthritis Outcome Score

	Mean Score at 15 y	Mean Score at 33 y	Mean Change	*P*	95% CI
Symptoms (n = 73)	87	82	5	.023	0.7 to 9.4
Pain (n = 73)	90	88	2	.314	−2.3 to 6.9
Activities of Daily Living (n = 72)	94	90	4	.060	−0.2 to 8.4
Sport and Recreation (n = 72)	77	68	9	.009	2.4 to 16.1
Quality of Life (n = 72)	74	71	3	.392	−3.8 to 9.5

#### Lysholm Knee Score

The mean Lysholm score between 15- and 33-year follow-up showed no essential change with 85 and 84 points, respectively (95% CI, –6.1 to 4.0; *P* = .752; n = 66).

#### Tegner Activity Score

The mean Tegner score decreased from before the injury to 15-year follow-up by 3 points (95% CI, −3.3 to −2.5; *P* < .001) and further to 33-year follow-up by 1 point (95% CI, −1.2 to −0.2; *P* = .004). From before the injury to 33-year follow-up, the mean score decreased by 4 points (95% CI, −4.1 to −3.2; *P* < .001; n = 99 at time of injury, n = 84 at 15-year follow-up, n = 77 at 33-year follow-up).

### Surgical Data

From the time of the ACL injury to 33-year follow-up, cumulative data showed that 25 (29%) patients were treated with ACLR (mean time to ACLR, 5 years), of whom 20 underwent ACLR in combination with treatment for a substantial meniscal injury (arthroscopic partial meniscectomy [APM] or suture repair). During follow-up, of these 20 patients, 8 were treated with meniscal repair in combination with late ACLR for those considered symptomatic (bucket-handle meniscal ruptures or unstable longitudinal ruptures in the red or red-white zone of the meniscus were repaired using inside-out sutures). In total, cumulative data showed that 48 patients (52%) underwent at least one event with meniscal surgery (APM or suture repair) with or without ACLR (mean time to APM/suture repair, 7 years) from the time of the injury until 33-year follow-up (Appendix Figure A1, available online).

Between 15- and 33-year follow-up, 3 patients were treated surgically for symptomatic OA (2 underwent TKR, and 1 underwent osteotomy [medial open wedge high tibial osteotomy]), whereas none had surgery before 15-year follow-up. One of the patients with TKR also underwent TKR in the non–ACL injured knee. Only a minority of patients (n = 13) had their surgical procedures performed after 15-year follow-up, and the mean time from the index ACL injury to further surgery was 22 years. Additional information on surgical procedures performed between 15- and 33-year follow-up is provided in Appendix Figure A1 (available online). Detailed data on additional knee injuries and surgical procedures performed from the time of the ACL injury to 15-year follow-up are described in the study by Neuman et al.^16^

## Discussion

Our main findings in this long-term follow-up study, at 33 years after an ACL injury in patients treated primarily without reconstruction, are that despite most patients (75%) having radiographic OA of the knee, only a minority (38%) were classified as having symptomatic OA (ie, radiographic OA in combination with a symptomatic knee according to cutoffs on the KOOS). Furthermore, the patients experienced overall acceptable subjective knee function but with a reduced level of activity compared with preinjury levels, an effect that can at least partially be explained by the normal adaptation of activities due to older age.

### Knee Function, Symptoms, and Activity Level at 33-Year Follow-up and Changes Over Time

The patients scored high on all KOOS subscales, indicating only minor symptoms and good knee function, even when compared with age-matched population-based reference values (Figure 2).^18^ We consider this a fair comparative reference, as the cohort was recruited randomly via mail from a population-based registry in the same region of Sweden as our study sample. One might have expected our KOOS scores to be inferior to reference values, but it is likely that the patients with ACL injuries in our study were more physically active, rendering higher KOOS scores compared with a “normal” population.

In a recent study by Kvist et al,^10^ patients with ACL injuries either treated with early ACL repair or treated primarily without ACL surgery were followed up for 32 to 37 years after the injury. Because our study included a similar cohort of primarily nonoperatively treated patients, we compared the KOOS Pain, Symptoms, Sport/Rec, and QOL subscores of our cohort with those of the cohort of Kvist et al. We noted that the patients in our study had clinically and statistically superior scores on all subscales, with up to 16 points better for both the Sport/Rec (95% CI, 7.3-25.2) and QOL (95% CI, 10.4-22.5) (see Appendix Table A6, available online). Furthermore, their patients who underwent early ACL repair presented even lower scores on the KOOS at final follow-up. Our cohort and the group treated primarily without reconstruction in the study by Kvist et al underwent structured rehabilitation for similar time periods after the ACL injury, but our cohort was also advised to avoid pivoting sports because they were considered a substantial risk for further knee injuries. Previous studies have shown a higher risk of reinjuries to the ACL^25^ and meniscus^15^ after returning to high-risk sports, which may, at least in part, explain our superior outcomes on the KOOS.

Between 15- and 33-year follow-up, only the KOOS Sport/Rec subscore showed a clinically significant mean deterioration, implying that most patients remained stable in their degree of pain, symptoms, activities of daily living, and quality of life between the 2 follow-up time points. This is in accordance with previous studies in which KOOS Sport/Rec and QOL subscores most often deteriorated over time.^12^ However, in our study, the KOOS QOL subscore showed only a slight decrease, which may indicate a superior outcome in our cohort.

For the Lysholm score, 65% of patients had a good or excellent outcome at 33-year follow-up, with a median score of 89 points. Compared with a study that followed patients for 20 years after either an operatively or nonoperatively treated ACL injury, our scores were similar to those of both groups (median, 86 and 89 points in operative and nonoperative groups, respectively).^23^

In our study, the Tegner score decreased from before the injury to final follow-up, which in a previous study has been suggested to be predominantly caused by age and lifestyle changes rather than knee symptoms.^6^ Most of the reduction occurred during the first 15 years of the study, which may represent patients reaching a plateau in activity levels between 15- and 33-year follow-up.

Except for the Tegner score, patients with radiographic OA scored worse on PROMs compared with patients without radiographic OA, and the KOOS and Lysholm scores were lower for patients with radiographic PF OA compared with patients with radiographic TF OA. As the group without radiographic OA was small, interpretations must be made with caution. However, these findings suggest a correlation between subjective knee function and symptoms with radiological findings. Furthermore, our results highlight the importance of evaluating both the TF and the PF joints for OA because the latter showed the best correlation with patients’ subjective knee function.

In our study, all the patients who underwent ACLR were noncopers regarding their ACL-deficient knees and had reconstruction after new episodes of the knee giving way. Giving-way episodes lead to an increased risk of meniscal lesions, which seems to be the most important risk factor for developing knee OA.^17^ Accordingly, we noted that more patients with radiographic OA underwent meniscal and ACL surgery from the time of the index injury to 33-year follow-up compared with patients without radiographic OA (49% vs 28%, respectively, and 24% vs 11%, respectively) (Appendix Table A5, available online). Thus, these results may indicate that it was mainly patients treated with late ACLR (mean, 5 years after injury) and meniscal surgery (mean, 7 years after injury) with subsequent OA development who were responsible for the lower KOOS and Lysholm scores. Identifying patients with a high risk for progressive knee instability and secondary meniscal injuries, who could benefit from early ACLR and other knee-protecting interventions, is a great challenge for the profession but could perhaps lower the development of radiographic OA in future patients with ACL injuries.

Some decrease in the Tegner score over time was expected, but knee symptoms causing problems during exercise may have lowered the activity level even further. A decrease in activity levels puts less stress on the knee, which might have resulted in higher scores on the other included PROMs. However, as our patients’ KOOS scores were similar to population-based reference values,^18^ with good Lysholm scores, we interpret these results as an indication that the patients were satisfied with their knee function, despite a low level of activity. All in all, our results indicate that, at least on a group level, patients can achieve subjectively acceptable knee function, but perhaps with a reduced activity level at >30 years after an ACL injury, when initially employing nonoperative treatment including physical therapy and activity modification. However, one must remember that the cohort of patients with ACL deficiency in this study is a mix of copers and noncopers in which noncopers were recommended late ACLR if they had insufficient knee stability. If the noncopers had been recommended to undergo further nonoperative treatment, the PROM scores would probably have been worse for these patients, subsequently lowering the mean scores for the whole cohort. Therefore, optional delayed ACLR should remain an alternative for noncopers if initial nonoperative treatment is undertaken.

### Radiographic and Symptomatic OA at 33-Year Follow-up

At 33-year follow-up, most patients had radiographic TF and/or PF OA. Compared with the primarily nonoperatively treated group in the study by Kvist et al,^10^ we had a higher prevalence of radiographic PF OA (30% vs 52%, respectively) but a lower prevalence of radiographic TF OA (71% vs 66%, respectively) and symptomatic OA (54% vs 38%, respectively). When further comparing these rates with those of the subgroup of Kvist et al allocated to early ACL repair, our cohort showed a higher prevalence of radiographic OA in both the PF (42% vs 52%, respectively) and TF (50% vs 66%, respectively) compartments but still a lower prevalence of symptomatic OA (44% vs 38%, respectively). However, the OA grading systems differed between the studies. Therefore, radiographic data cannot be directly compared, whereas the symptomatic OA comparisons are probably more reliable. With this in mind, our study seems to show long-term beneficial results on symptomatic OA, but not necessarily on radiographic OA, compared with both surgically and nonsurgically treated ACL-injured knees in other studies. Furthermore, our findings emphasize the importance of studying both radiographic and symptomatic outcomes, as a significant prevalence of symptomatic OA may otherwise be overestimated.

### Surgical Data

At 33-year follow-up, 29% of patients had undergone ACLR, which is lower compared with the primarily nonoperatively treated group (40%) in the study by Kvist et al.^10^ Furthermore, we had a lower prevalence of TKR (2% vs 7%, respectively).^10^ Approximately half of the patients in our study underwent meniscal surgery by 33-year follow-up, which may represent poor capacity in maintaining meniscal integrity, which would be in accordance with previous research reporting a protective role of ACLR on the meniscus.^11,19^ However, our prevalence of secondary meniscectomy is lower compared with that of other long-term follow-up studies. One study found that 76% of patients with ACL injuries treated nonoperatively had a secondary meniscal injury at 20 years after the index injury,^23^ and one study reported that 95% of high-level athletes with ACL injuries treated without reconstruction underwent meniscectomy up to 20 years after the injury.^15^

### Limitations

This study includes some limitations. First, almost 1 year elapsed from the completion of questionnaires until radiography for some patients because of restrictions during the COVID-19 pandemic. Second, the applied definition of symptomatic knee OA is not based on a validated classification system, which should be taken into consideration in the interpretation of the results.

Third, all our patients had diagnostic arthroscopic surgery performed shortly after the ACL injury, during which hemarthrosis was washed out. The inflammatory response caused by hemarthrosis may drive the development of OA^5^; thus, early arthroscopic surgery might have been beneficial for our cohort. Because arthroscopic surgery is not part of contemporary practice after an ACL injury, this might have contributed to better subjective knee function for our cohort than would be seen in patients outside of this study.

Fourth, all reconstrcuted patients in this study underwent late ACLR, and the interpretation of the results must be considered in light of these drawbacks. However, treatment recommendations are constantly evolving, and long-term follow-ups will always be associated with such issues. The intention of the study was to examine how patients with ACL injuries manage without reconstruction, which we believe will provide important information on how treatment recommendations should develop in the future.

Fifth, statistical comparisons were performed between our KOOS scores and the KOOS scores of 2 different cohorts provided by other studies. The intention of the statistical comparisons was to provide an opportunity to compare our scores with the scores of other cohorts to compensate for the fact that we did not have a control group. However, these comparisons include many flaws, as the statistical comparisons are unadjusted and do not consider confounding factors or selection bias. Because of this complexity, the interpretation of these comparisons should be made cautiously.

Finally, the patients included at 15-year follow-up who were lost to 33-year follow-up had a 3–percentage point higher prevalence of meniscal surgery, which may have skewed our results to somewhat higher PROM scores and a lower prevalence of radiographic OA. Also, we have data only on those patients who underwent meniscal surgery, whereas the actual number of meniscal injuries might be greater.

## Conclusion

In this study, we found acceptable subjective knee function with a low prevalence of symptomatic OA, despite a high prevalence of radiographic OA, at >30 years after an ACL injury treated primarily without reconstruction. Furthermore, 71% of patients were managed without late ACLR, and the prevalence of surgery because of OA was low. The patients may, however, have been exposed to an increased risk of meniscal injuries. All patients were advised to avoid cutting motions or pivoting of the knee, and most patients had a decrease in activity levels until final follow-up. However, symptoms and knee function deteriorated only slightly until 33 years of follow-up. Altogether, our results may indicate that physical therapy and early activity modification with optional delayed ACLR might be a good treatment option for patients with low to moderate activity levels.

## Supplemental Material

sj-pdf-1-ajs-10.1177_03635465231214423 – Supplemental material for Outcomes >30 Years After Initial Nonoperative Treatment of Anterior Cruciate Ligament Injuries Click here for additional data file. Supplemental material, sj-pdf-1-ajs-10.1177_03635465231214423 for Outcomes >30 Years After Initial Nonoperative Treatment of Anterior Cruciate Ligament Injuries by Clara Hellberg, Ioannis Kostogiannis, Alexandros Stylianides and Paul Neuman in The American Journal of Sports Medicine
