# Supplementary material for: Outcomes >30 Years After Initial Nonoperative Treatment of Anterior Cruciate Ligament Injuries
Source: Am J Sports Med. 2024 Jan 9;52(2):320–9. doi: 10.1177/03635465231214423 (PMC10838478; doi:10.1177/03635465231214423)
Supplement: sj-pdf-1-ajs-10.1177_03635465231214423 – Supplemental material for Outcomes >30 Years After Initial Nonoperative Treatment of Anterior Cruciate Ligament Injuries [file sj-pdf-1-ajs-10.1177_03635465231214423.pdf]

Appendix

Figure A1. Flow chart of the cumulative incidence of surgical procedures up to 15- and 33-year follow-up, and the incidence during the follow-ups, where a total of 13 re-operations occurred.

N at 15-year follow-up: 93

N at 33-year follow-up: 81

Nota bene, the cumulative incidence at 33-year follow-up %-values are adjusted to more than 81 followed-up patients for each studied event. Meniscus injury refers to a substantial meniscal injury treated with arthroscopic partial meniscectomy (APM) or suture repair. ACLR = Anterior cruciate ligament reconstruction. MFX = microfracture procedure for deep knee cartilage defect. KS = knee synovectomy. TKR = total knee replacement.

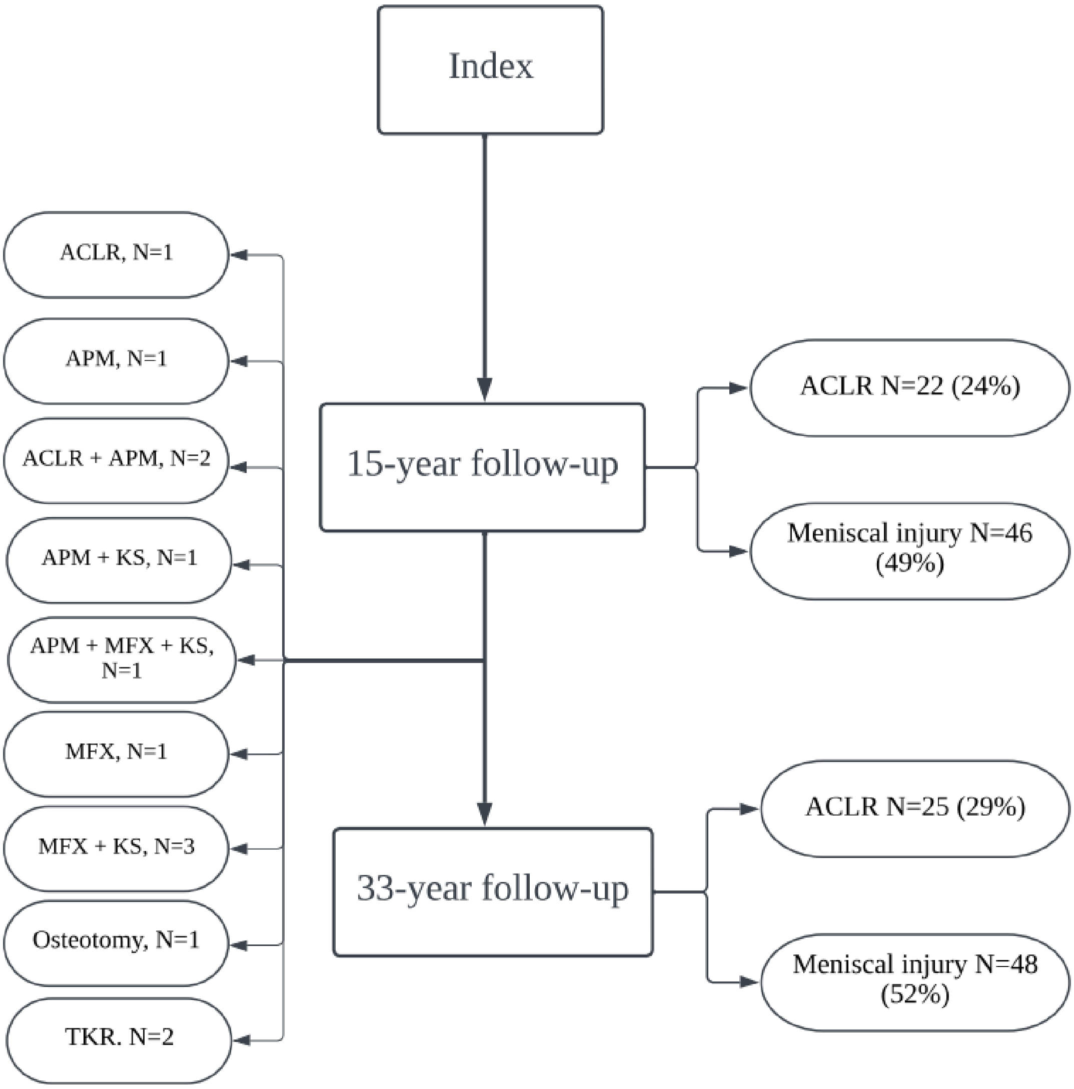

Table A1. Student's t-test for differences in KO OS scores at 33-year follow-up and age-representative population-based reference values (55-74 years).
N at 33-year follow-up: Symptoms 78, Pain 78, ADL 78, Sport/Rec 77, QOL 77
N for reference values: Symptoms 173, Pain 173, ADL 173, Sport/Rec 171, QOL 173

KOOS subscale	Mean KOOS 33-year follow-up	Mean KOOS reference values	Mean difference	p-value	95% CI
Symptoms	82	83	-1	0.805	-6.4 – 4.9
Pain	88	83	5	0.072	0.5 – 10.7
ADL	91	82	9	0.003	2.9 – 14.4
Sport/Rec	68	67	1	0.769	-7.6 – 10.2
QOL	71	74	-2	0.526	-9.9 – 5.1

Table A2. Linear regression analyses for all PROMs vs ROA yes/no (adjusted for sex, age, and BMI at the 33-year follow-up) of the radiographed patients at 33-year follow-up. The differences in points are for patients with ROA compared with patients without ROA.
N with ROA: 52
N without ROA: 18

		TF and/or PF ROA	
	Difference in points	p-value	Confidence interval
KOOS symptoms	-8.2	0.144	-19.2 - 2.9
KOOS pain	-11.0	0.023	-20.4 – (-1.6)
KOOS ADL	-7.3	0.134	-17.0 - 2.3
KOOS Sport/Rec	-23.5	0.008	-40.8 – (-6.3)
KOOS QOL	-16.6	0.019	-30.4 – (-2.8)

		TF ROA	
	Difference in points	p-value	Confidence interval
KOOS symptoms	-8.7	0.081	-18.4 - 1.1
KOOS pain	-9.6	0.026	-18.0 – (-1.2)
KOOS ADL	-6.1	0.168	-14.6- 2.6
KOOS Sport/Rec	-19.8	0.013	-35.3 – (-4.3)
KOOS QOL	-14.1	0.027	-26.5– (-1.6)

		PF ROA	
	Difference in points	p-value	Confidence interval
KOOS symptoms	-12.6	0.008	-21.8- (-3.5)
KOOS pain	-10.6	0.010	-18.6– (-2.6)
KOOS ADL	-9.0	0.031	-17.2 – (-0.8)
KOOS Sport/Rec	-20.0	0.010	-35.0– (-5.0)
KOOS QOL	-12.9	0.037	-25.0 – (-0.8)

		TF and/or PF ROA	
	Difference in points	p-value	Confidence interval
Lysholm	-10.9	0.031	-20.8 – (-1.0)

		TF ROA	
	Difference in points	p-value	Confidence interval
Lysholm	-9.4	0.039	-18.2 – (-0.5)

		PF ROA	
	Difference in points	p-value	Confidence interval
Lysholm	-11.2	0.010	-19.6 – (-2.7)

		TF and/or PF ROA	
	Difference in points	p-value	Confidence interval
Tegner	0.2	0.630	-0.7 - 1.1

		TF ROA	
	Difference in points	p-value	Confidence interval
Tegner	0.1	0.845	-0.7 - 0.9

		PF ROA	
	Difference in points	p-value	Confidence interval
Tegner	0.3	0.455	-0.5 - 1.1

Table A3. Number and prevalence of patients with TF, PF, or TF and/or PF ROA stratified by age groups.

Age group (years)	TF ROA, Number (%)	PF ROA, Number (%)	TF and/or PF ROA, Number (%)
40-49	1 (25)	1 (25)	1 (25)
50-59	25 (61)	19 (46)	29 (71)
60-69	14 (70)	11 (55)	17 (85)
70-79	8 (100)	7 (88)	8 (100)

Table A4. Number, prevalence, and confidence interval (%) of the patients with or without ROA in combination with being symptomatic or non-symptomatic on KOOS at 33-year follow-up.

Combination ROA + KOOS symptomatic/non-symptomatic	Number (%)	Confidence interval %
TF and/or PF ROA + symptomatic KOOS	28 (38)	28-50
TF and/or PF ROA + non-symptomatic KOOS	27 (37)	27-49
No ROA + symptomatic KOOS	5 (7)	3-15
No ROA + non- symptomatic KOOS	13 (18)	11-28
TF ROA + symptomatic KOOS	26 (36)	26-47
TF ROA + non- symptomatic KOOS	22 (30)	21-42
No TF ROA + symptomatic KOOS	7 (10)	4-19
No TF ROA + non- symptomatic KOOS	18 (25)	16-36
PF ROA + symptomatic KOOS	23 (32)	22-43
PF ROA + non- symptomatic KOOS	15 (21)	13-31
No PF ROA + symptomatic KOOS	10 (14)	7-24
No PF ROA + non- symptomatic KOOS	25 (34)	24-46

Table A5. Patient characteristics and surgery data (ACLR, meniscal surgery, or no surgery if not having had ACLR or meniscal surgery) divided by TF and/or PF ROA yes/no, of radiographed patients at 33-year follow-up.

N with ROA: 55

N without ROA: 18

	TF and/or PF ROA at 33-year follow-up	No ROA at 33-year follow-up
Age, mean (SD) years [range]	60 (8) [48-76]	54 (5) [48-64]
Women, N (%)	24 (44)	8 (44)
Body mass index, mean (SD) kg/m2	26 (4), n=53	27 (4), n=17
ACLR, N (%)	13/55 (24%)	2/18 (11%)
Meniscal surgery, N (%)	27/55 (49%)	5/18 (28%)
No surgery, N (%)	25/55 (45%)	13/18 (72%)

Table A6. Student’s t-test for mean differences in KOOS scores at 33-year follow-up and the ACL-injured group allocated to non-surgical treatment in the study by Kvist et al.[10] Mean difference (higher points for our cohort), p-value, and confidence intervals.
N at 33-year follow up: Symptoms 71, Pain 78, Sport/Rec 77, QOL 77
N in Kvist et al.: Symptoms 89, Pain 89, Sport/Rec 89, QOL 89

KOOS subscale	Mean difference (points) between our cohort vs. Kvist et al.	p-value	Confidence interval
Symptoms	11	<0.001	4.9-17.4
Pain	8	0.003	2.8-13.9
Sport/Rec	16	<0.001	7.3-25.2
QOL	16	<0.001	10.4-22.5
